# Antimicrobial Resistance Creates Threat to Chimpanzee Health and Conservation in the Wild

**DOI:** 10.3390/pathogens10040477

**Published:** 2021-04-14

**Authors:** Michele B. Parsons, Dominic A. Travis, Elizabeth V. Lonsdorf, Iddi Lipende, Deema Elchoufi, Baraka Gilagiza, Anthony Collins, Shadrack Kamenya, Robert V. Tauxe, Thomas R. Gillespie

**Affiliations:** 1 Department of Environmental Sciences and Program in Population Biology, Ecology, and Evolutionary Biology, Emory University, Atlanta, GA 30322, USA; zcp9@cdc.gov (M.B.P.); delchou@emory.edu (D.E.); 2 Department of Environmental Health, Rollins School of Public Health, Emory University, Atlanta, GA 30322, USA; 3 Centers for Disease Control and Prevention, Atlanta, GA 30329, USA; rvt1@cdc.gov; 4 College of Veterinary Medicine, University of Minnesota, Minneapolis, MN 55108, USA; datravis@umn.edu; 5 Department of Psychology, Franklin and Marshall College, Lancaster, PA 17603, USA; Elizabeth.lonsdorf@fandm.edu; 6 The Jane Goodall Institute, Kigoma, Tanzania; lipende2001@yahoo.co.uk (I.L.); bgilagiza@janegoodall.or.tz (B.G.); acollins@janegoodall.or.tz (A.C.); skamenya@janegoodall.or.tz (S.K.)

**Keywords:** AMR, Gombe, one health, primate, sulfonamides, Tanzania, tetracycline, zoonoses

## Abstract

Infectious disease is recognized as the greatest threat to the endangered chimpanzees made famous by the groundbreaking work of Dr. Jane Goodall at Gombe National Park (GNP), Tanzania. The permeable boundary of this small protected area allows for regular wildlife–human and wildlife–domestic animal overlap, which may facilitate cross-species transmission of pathogens and antimicrobial resistance. Few studies have examined the prevalence of antimicrobial resistance in wild ape populations. We used molecular techniques to investigate the presence of genes conferring resistance to sulfonamides (often used to treat diarrheal illness in human settings in this region) and tetracycline (used in the past—though much less so now) in fecal specimens from humans, domestic animals, chimpanzees, and baboons in and around GNP. We also tested stream water used by these groups. Sulfonamide resistance was common in humans (74%), non-human primates (43%), and domestic animals (17%). Tetracycline resistance was less common in all groups: humans (14%), non-human primates (3%), and domestic animals (6%). Sul resistance genes were detected from 4/22 (18%) of streams sampled. Differences in sul gene frequencies did not vary by location in humans nor in chimpanzees.

## 1. Introduction

Antimicrobial agents have saved millions of lives worldwide, but the global emergence of resistance is compromising their efficacy. Antimicrobial usage in both human and domestic animal populations provides selective pressure for the spread of resistance [1,2]. In many parts of the world, unregulated over-the-counter sales of antimicrobial drugs have facilitated unmitigated, frequent, and inappropriate use in both humans and animal agriculture [3]. Resistant strains also emerge as a result of horizontal gene transfer of mobile elements carrying resistance genes [4].

Wildlife are not typically administered antimicrobial drugs but can acquire antimicrobial-resistant bacteria through contact or shared resources with humans, domestic animals, and the environment. Proximity to humans has been associated with a higher prevalence of antimicrobial resistance (AMR) in some wildlife populations, and exposure to antibiotics from anthropogenic sources affects AMR in the gut bacteria of wild animals [5,6,7,8]. In addition, wildlife may serve as sentinels of emerging resistant bacterial pathogens or genes in the environment [6,9,10].

Few studies have examined the impact of antimicrobial resistance in ape populations. In Bwindi Impenetrable National Park, Uganda, AMR was higher from gut bacterial isolates of gorillas that had greater shared habitat with humans and livestock [8]. A separate study in Kibale National Park found that bacteria from chimpanzees were genetically more similar to those in humans working in chimpanzee-directed research and tourism than humans in the local population [11]. This research suggests that shared habitat and human interaction with wild apes facilitate bacterial transmission and antimicrobial resistance. Further studies are needed to understand the burden and risks for AMR in natural ape populations.

Infectious disease is recognized as the leading cause of mortality in the well-studied population of chimpanzees (*Pan troglodytes schweinfurthii*) of Gombe National Park (GNP), Tanzania [12]. The park lies on the eastern shore of Lake Tanganyika in western Tanzania, with villages flanking the north and south. The park’s small size (35 km^2^) and porous boundary results in opportunities for wildlife–human and wildlife–domestic animal overlap [13,14]. This interface creates exceptional opportunity for microorganism transfer between local humans, tourists, domestic animals, and wildlife [15,16,17].

**Figure 1:**
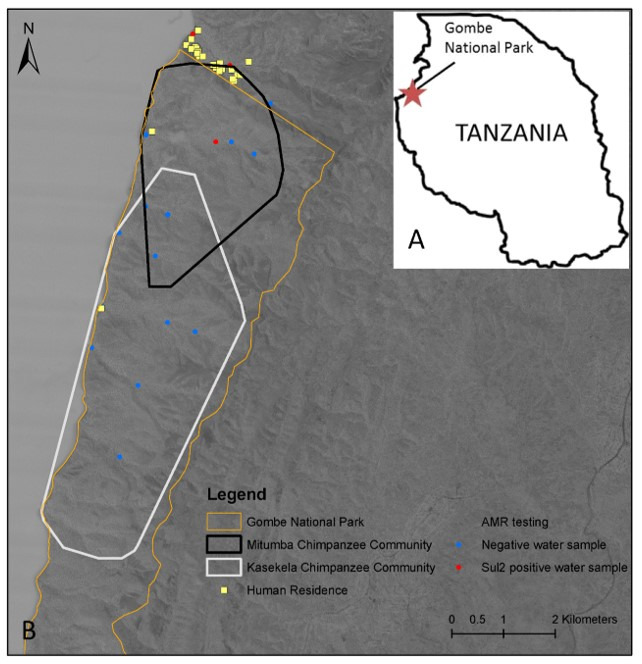
The study site. (**A**) Location of Gombe National Park in Tanzania. (**B**) Home ranges for the chimpanzee (*Pan troglodytes*) groups studied; Mitumba and Kasekela and water sampling locations of park and village streams. Yellow squares are individual households (Mwamgongo) or the camp.

The park (Figure 1) contains two habituated chimpanzee groups with inherently different degrees of human contact. Mitumba (estimated population size (*n*)~25 individuals), has a constricted range (~5 km^2^) within one stream valley with the boundary of the park and the village of Mwamgongo to the north (*n*~5000 inhabitants) and the Kasakela chimpanzee community to the south [18]. In contrast, Kasekela (estimated population size (*n*)~65 individuals), has a substantially larger home range (~16 km) in the center of the park where tourists, researchers, and park employees (*n*~100) interact with primates to some degree on a daily basis [19]. There is limited interaction between the territorial chimpanzee communities.

In exceptional circumstances, morbidly sick chimpanzees in GNP were administered antimicrobials—usually through the provisioning of treated fruit. From 2003–2010, antimicrobials were administered on only two occasions to a single individual chimpanzee each time [19]. Therefore, antimicrobial therapy in Gombe chimpanzees is a rare occurrence, thus detection of AMR suggests bacterial spillover from humans and/or domesticated animal populations.

The aim of our research was to quantify the prevalence of resistance genes in human, domestic animal, and non-human primate (NHP) populations in the Greater Gombe Ecosystem (GGE), and examine patterns of overlap between groups. We measured the prevalence of genes coding for resistance to the most frequently used antimicrobial drugs in this area, sulfonamides (often used to treat diarrheal illness in human settings in this region) and tetracycline (often used in the past—though less so now) [20,21]. We tested for genes, *sul1*, *sul2*, *tetA*, *tetB* to evaluate them as proxies for bacterial spillover transmission and to identify potential risk factors for the presence of AMR in chimpanzees. These genes are common acquired determinants for resistance in the gut microbiome [22,23,24] with a limited spectrum of intrinsic resistance activity [25]. We predicted that sulfonamide resistance would be higher than tetracycline resistance in all groups, that humans would have the highest prevalence of sulfonamide resistance of any group and that the Mitumba chimpanzees would have greater sulfonamide resistance when compared to Kasekela chimpanzees because of more opportunity for contact with a densely populated human setting.

## 2. Results

### 2.1. Descriptive Statistics of All Individuals

Categorical prevalence rates for sulfonamide and tetracycline resistance in all human and animal populations are presented in Table 1; specific gene resistance prevalence is presented in Table 2. Overall, prevalence (%) was highest in humans, followed by domestic animals, and then NHPs. Sulfonamide resistance was found at a much higher frequency than tetracycline resistance in all categories: humans (McNemar’s 4.99; *p* < 0.001), NHPs (McNemar’s 3.40; *p* < 0.05), and domesticated animals (McNemar’s 10.68; *p* < 0.001). This finding also held for chimpanzees (McNemar’s 2.64; *p* < 0.05) but was not observed in goats (McNemar’s 0.800; *p* = 0.371) or baboons (McNemar’s 0.363; *p* < 0.546) when examining individual species. Small sample sizes limited evaluation of sulfonamide versus tetracycline prevalence in sheep or dogs.

Sulfonamide resistance in humans was comparable across all locations: Mitumba (81.3%), Kasekela (78.7%), and Mwamgongo (69.1%). The two chimpanzee communities also had similar frequencies of sulfonamide resistance: Mitumba (42.8%) and Kasekela (50%). Sulfonamide prevalence was higher in dogs (71.4%) than goats (10%, Fisher’s Exact Test *p* = 0.001) or sheep (23%, Fisher’s Exact Test *p* = 0.001). Sulfonamide resistance was more prevalent in humans than chimpanzees (*X*^2^ =16.734, df = 1, *p* < 0.05) and domestic animals (*X*^2^ = 80.771, df = 1, *p* < 0.05). Sulfonamide resistance was detected more frequently in chimpanzees than domestic animals (*X*^2^ = 18.426, df = 1, *p* < 0.05).

### 2.2. Diversity of Sulfonamide and Tetracycline Genes in Fecal Specimens

The frequency of sulfonamide and tetracycline resistance genes detected in all human and animal populations is presented in Table 2. In every group, *sul2* was detected more frequently than *sul1*. Humans positive for *sul2* were more likely to also be positive for *sul1* (40%) than those positive for *sul1* were to have *sul2* positivity (7%) (McNemar’s 5.88; *p* < 0.001). The same trend was observed in chimpanzees: 35% with *sul2* positivity also had *sul1*, but only 1% for those positive for *sul1* to also be positive for *sul2* (McNemar’s 6.81, *p* < 0.001) and domestic animals: 8% with *sul2* also contained *sul1* as compared to 2% for those with *sul1* to have *sul2* (McNemar’s 20.66; *p* < 0.001). *Sul* gene frequencies in baboons were not significantly different (McNemar’s 0.449, *p* > 0.05).

*TetA* and *tetB* genes were detected at differing frequency in the human and animal populations. *TetB* was detected more frequently than *tetA* in humans (McNemar’s 2.223, *p* < 0.001). Only *tetB* (6%) was detected in chimpanzees. Tetracycline resistance was not detected in baboon specimens. Both *tetA* and *tetB* genes were detected in specimens from goats and dogs.

### 2.3. Stream Water Sampling

Sulfonamide resistance genes were found in 4 (18%) of 22 stream samples; all four were permanent streams. No tetracycline resistance genes were detected. Detection of sulfonamide resistance genes did not vary by season (*X*^2^ = 0.512, df = 1, *p* > 0.05). Positive sampling sites were Mitumba and Mpemba streams, which run continuously. Mitumba stream is in the park and runs through Mitumba camp. Mpemba stream runs through Mwamgongo village (Figure 1).

### 2.4. Risk Factors for Sulfonamide Resistance

Neither chimpanzee age, sex, nor community of residence was associated with sulfonamide resistance. There was no seasonal variation in sulfonamide resistance in humans (*X*^2^ = 0.251, *p* > 0.05) or chimpanzees (*X*^2^ = 0.006, *p* > 0.05).

## 3. Discussion

Genes encoding sulfonamide resistance were detected at much higher frequencies than tetracycline resistance in all groups. Sulfonamide resistance in humans (74.3%) was much higher than tetracycline resistance (13.9%). There is no existing data regarding antimicrobial resistance in any of the studied communities and thus no “expected” levels for comparison. In addition, this cross-sectional study was not designed to establish baseline levels of resistance, but to provide a snapshot of the situation in order to develop further hypotheses. However, there is some limited data on local usage, which may infer risk. A review of drug dispensary reports in Mwamgongo (clinic and hospital) in association with the health clinical officer reveals that antimicrobials are available without prescription. Co-trimoxazole (sulfamethoxazole-trimethoprim) is cheap and frequently purchased (bottle cost: 5000 Tanzanian Shillings (TSH); 2.29 USD) in both locations, potentially providing the selective pressure to maintain sulfonamide resistance in the human population. On the other hand, tetracycline is rarely administered due to its high cost (bottle cost: 25,000 TSH; 11.43 USD) and more limited availability (village clinic). A recent study in Uganda found that antimicrobial resistance phenotypes were similar in people and animals, and the percentage of resistant isolates decreased with increasing local price of the antibiotic [26]. The domestic animals had relatively low sulfonamide resistance (16.9%), except for dogs, which had frequencies comparable to humans (71.4%), which could be explained by (i) small sample size and (ii) a more intimate relationship with humans than livestock. The village animal health officer reported that domestic animals are rarely treated with antimicrobials.

Between 40–50% of NHPs were positive for sulfonamide resistance, but less than 6% were positive for tetracycline resistance. Although background rates in this park are unknown, the high sulfonamide resistance for an untreated population of NHPs suggests anthropogenic acquisition of these genes. These results parallel findings from Uganda where bacterial isolates from humans (35%), livestock (27%), and gorillas (17%) in and around Bwindi Impenetrable National Park were clinically resistant to at least one antibiotic used by the resident human population and AMR in gorilla bacteria was positively associated with human overlap [8]. Similarly, the majority of humans (81.6%) and 4.4% of chimpanzees in and around Kibale National Park in Uganda had at least one isolate resistant to locally available antibiotics. Isolates recovered from humans and chimpanzees also showed greater resistance to five locally available antibiotics than to Ceftiofur, an antibiotic not available in the region [11]. The data suggest human–wildlife interactions and shared environment can facilitate bacterial and AMR transmission [11].

Although we had expected the Mitumba chimpanzees to have greater sulfonamide resistance than Kasekela chimpanzees, we found no difference. Thus, we found no evidence that proximity to Mwamgongo itself provides a greater risk during this sample period. This was surprising given that Mitumba chimpanzees are a smaller community, with a narrower natural range that borders the dense human population of Mwamgongo. Perhaps more intimate, regular, contact with the human environment regardless of the size of the human population is the strongest risk factor.

Kasekela was the first chimpanzee group to be habituated, and human acceptance may increase camp visits and food theft [19]. While efforts are made in the camp to secure food and contain human waste; these systems are not perfect and may result in environmental contamination. NHPs and other forest animals may come in contact with food or human feces, placing them at risk to acquire enteric bacteria and associated plasmid-mediated resistance genes via the fecal–oral route [13]. In a study in Amboseli National Park, Kenya, baboons who had daily contact with unprocessed human refuse had a higher proportion of AMR bacteria compared to those baboons living in undisturbed habitat [27]. Although the human population in Kasekela is far smaller than Mwamgongo, the residents of this camp consist of transient tourists, researchers, and park employees. These groups have a more reliable income and can afford to purchase a broader variety of antimicrobials compared to village residents [1,28]. Park staff may feel pressured to take antimicrobials or antimalarial medication when they feel ill as symptomatic researchers and field staff are restricted from work via a mandatory quarantine process. These explanations are consistent with findings from Bwindi Impenetrable National Park, Uganda, where local antimicrobial use in park staff associated with the frequency and class of AMR genes recovered from gorillas [8].

Direct contact with humans is not the only source of AMR for NHPs. Although sample sizes were small, the high prevalence of *sul* genes in dogs (77.7%) may increase the risk for AMR transmission to NHPs. Dogs utilize the park and frequent crop fields in the village that are regularly raided by chimpanzees [14]. Sulfonamide antimicrobials have a high excretion rate in humans and animals [29]. They can accumulate in the environment and have been shown to leach from the soil into groundwater systems [30]. The two locations where *sul2* genes were detected are used by humans in the camp (Mitumba stream) and village (Mwamgongo stream) for bathing, washing of food and utensils, and by NHPs and domesticated animals for drinking water. A study limitation was that it was not logistically feasible to collect and filter large volumes of water in the field which could increase environmental detection of AMR genes. Therefore, our negative results do not assure that AMR genes are not circulating in the aquatic system.

A case-control study in Kruger National Park, South Africa showed that impala drinking from antimicrobial infected rivers were at a higher odds risk for infection with AMR gut flora relative to their unexposed counterparts [31]. A study in Gabon analyzed the phylogenetic and antimicrobial profile of *E. coli* strains from humans, livestock, and wildlife, including gorillas, and found no evidence of transmission of antibiotic resistant *E. coli* strains from humans to gorillas [32]. The strains were distinct suggesting that horizontal gene transfer or naturally acquired resistance might be responsible instead of bacterial transmission from humans. 

Across all groups, there was a higher prevalence of *sul2* detected, and the presence of *sul2* correlated with the concurrent presence of *sul1*. Human studies report similar trends with *sul2* prevalence higher than *sul1*, and in the absence of sulfonamide use there is no remarkable decline in community resistance [33]. These genes have distinct transfer mechanisms, which may explain the variation in observed frequencies. The *sul1* gene is typically linked to other resistance genes in class 1 integrons; however, *sul2* is frequently located on small non-conjugative plasmids or large transmissible multi-resistance plasmids, carrying genes to a number of other antimicrobials that may be under positive selection [34,35]. The frequency of *tetA* and *tetB* genes was low across all groups. Tetracycline resistance is widespread among diverse bacterial species with varying modes of action. Some genes, such as *tetA* and *tetB*, encode for efflux pumps that remove the drug from the cell, while others provide ribosomal protection of the drug binding site [36]. These genes are typically present on conjugative transposons that are prone to horizontal gene transfer between species. While *tetA* and *tetB* are common genes that confer resistance to tetracycline, we need to interpret the findings of low tetracycline resistance in this study population with caution. There are other tetracycline resistance genes, notably *tetM*, and *tetO*, that we did not survey and could be present, such that the proportion and distribution of resistance may look different. Non-culture based approaches are also limited in that we are detecting resistance genes without knowledge of their bacterial origin, or if the species is viable in the microbiome. Further studies would be recommended to better understand antimicrobial resistance in this system.

Sulfonamide resistance among humans aligned with frequently reported use of this drug to treat gastroenteritis and other diarrheal ailments by villagers and camp residents. These findings highlight potential treatment concerns, where a drug or dosage was ineffective or an antimicrobial was not necessary. In this system, domesticated animals, chimpanzees, and baboons are not routinely treated with antimicrobials. It is worrisome that resistance to commonly used antimicrobials in the human population already exists in NHPs and behooves testing recovered gastrointestinal bacteria from these groups for AMR. Should a Gombe chimpanzee need antimicrobial treatment, resistance testing would be essential to choose an appropriate drug.

Our findings demonstrate that humans and NHPs in the GGE had high levels of sulfonamide resistance during this sampling period. Selective pressure for sulfonamide resistance in humans is likely maintained by the frequent, unregulated use of co-trimoxazole. Regular opportunity for contact with human environments is a possible route exposing untreated NHPs to drug-resistant bacteria and antimicrobial resistance genes. Habitat reduction from human encroachment will continue to increase potential contact with humans and increase the risk for AMR and disease transmission. To minimize the spread of disease and resistance in both human and animal populations, practical and sustainable public health recommendations are needed, including promoting safe water and improved sanitation, AMR monitoring, and antibiotic stewardship.

## 4. Materials and Methods

### 4.1. Study Site

Gombe National Park, Tanzania (4°41’59.97”S, 29°36’59.96”E), is a 35 km^2^ forest reserve located 1500 m above sea-level extending to Lake Tanganyika on the west and villages to the north and south [13] (Figure 1). Wildlife and village or camp residents share some park resources. For instance, residents use the lake and streams for bathing, washing clothes, and household/cooking utensils, while serving as the shared water source of humans, baboons, and chimpanzees [13]. The park border is permeable; villagers and their animals enter the park, and chimpanzees and baboons have been reported outside the park, sometimes to raid crop fields in Mwamgongo village, leaving feces on human food sources [14].

### 4.2. Sample Collection Period

Fecal samples were collected in 2010 during dry (July 1–August 15) and wet (November 1–December 15) seasons. Fecal specimens were collected from humans (*n* = 187), domestic animals: dog (*Canis lupus*, *n* = 7), goat (*Capra hircus*, *n* = 69,) and sheep (*Ovis aries*, *n* = 13), and NHPs: chimpanzees (*n* = 75) and baboons (*Papio anubis*, *n* = 47). Human subjects were either residents of Mwamgongo village or Gombe National Park; tourists were not sampled. Twenty-five village households with domesticated animals were randomly selected for study enrollment [14]. Individually recognized baboons (*Papio anubis*) were opportunistically sampled during these two collection periods. Chimpanzees, in both Mitumba and Kasekela, have well studied life histories and were sampled during the course of routine observational health monitoring [19].

### 4.3. Specimen Collection and Transport

All fecal specimens were freshly voided and transferred aseptically to a screw cap plastic vial containing a 2.5% potassium dichromate solution (Fisher Scientific, Pittsburgh, PA). Enrolled village and park residents were provided specimen cups with instructions for stool collection. Domesticated animal specimens were collected aseptically by a village veterinary officer. For NHP samples, samples were non-invasively collected from identified individuals as part of observational health monitoring. For NHP specimens, care was taken to avoid the collection of soil, foliage, or water contaminants, by transferring the interior and top most portion of stool to a collection cup using a sterile wooden spatula or swab and avoiding the collection of fecal material in contact with the ground. Each vial was labeled with a unique identification number, and date of collection. NHP samples were also labeled with the name of the observer, location, and animal name. Samples were sealed with Parafilm (Pechiney Plastic Packaging, Chicago, IL), refrigerated, and shipped at 4 °C to Atlanta, Georgia, USA.

### 4.4. Water Sampling

We examined water samples from six annual (dry and wet seasons) and two seasonal (wet only) streams within the GGE that are used by these groups. Water samples were collected from low, middle, and high points of six permanent streams and two seasonal (wet only) streams, using sterile gauze Moore swabs immersed overnight for 18–24 h in flowing water. Swabs were transferred to sterile containers containing RNAlater (stored at ambient temperature) or tryptic soy broth with 20% glycerol (stored in a cryogenic storage dewar) prior to shipment to Atlanta, GA, USA. GPS coordinates were obtained using a GPSmap 60 CSx from Garmin (Garmin International Inc. Olathe, KS) for each collection point to assist in identifying locations for repeat sampling and if necessary, to assign sampled streams to watersheds associated with specific human or chimpanzee groups.

### 4.5. Detection of Resistance Genes to Sulfonamide and Tetracycline

Nucleic acid was extracted from 200 µL aliquots of each fecal specimen, water sample and positive control sample using the FastDNA^®^ SPIN Kit for Soil (MP Biomedicals, LLC, Solon, OH, USA) following the methods described [37]. Total nucleic acid extractions were stored at −20 °C for working use and archived at −80 °C. A multiplex polymerase chain reaction (PCR) was used to detect the presence of genes for sulfonamide resistance (*sul1*, *sul2*) and tetracycline resistance (*tetA*, *tetB*) genes [38]. Each 25 µL PCR reaction consisted of 17.25 µL sterile distilled water, 2.5 µL of 10X PCR buffer, 2.5 µL of deoxynucleoside triphosphates (2.5 mM [each] dATP, dTTP, dCTP, dGTP), 0.5 µL of 25µM primer mix, 0.25 µL *Taq* polymerase and 2 µL of template in a 1.5 mL microcentrifuge tube. Sterile distilled water was used as a negative control, positive control strains were kindly provided by the Enteric Diseases Laboratory Branch, CDC, (Atlanta, GA, USA). *E. coli* strain DH0032 was used as the positive control for *sul1,* and *E. coli* strain DH3507 was the positive control for *sul2*. *Salmonella* strains 2013K-0573 and 2013K-1023 were positive control strains for *tetA* and *tetB* genes respectively. Ten microliters of the PCR product were electrophoresed on 1.5% SeaKem^®^ LE Agarose (Lonza, Rockland, ME, USA) gels. Gels were stained by adding 3 µL of ethidium bromide to the gel and 2 µL to the running buffer. Gel images were captured under UV exposure using a Gel Doc illumination system (Bio-Rad, Hercules, CA, USA).

### 4.6. Data Analysis

Statistical analyses were performed with SPSS Statistics version 20 (International Business Machines, Armonk, NY, USA). A chi-square test of independence was used to compare sulfonamide resistance by group, species, or season. 95% confidence intervals were calculated for all cross-tabulations with a two-tailed significance set at 0.05 for all comparisons. In instances where cells contained less than five values, Fisher’s exact tests were used to calculate *p*-values. McNemar’s test was used to compare sulfonamide versus tetracycline prevalence and *sul2* versus *sul1* frequencies. To identify association between chimpanzee population characteristics, and sulfonamide resistance we used a generalized estimating equation (GEE) method with exchangeable working correlation structure to account for repeat sampling of individuals in both dry and wet seasons. The Huber–White sandwich variance estimation technique was used to calculate confidence intervals (CI).

## Figures and Tables

**Table 1:** Frequency (%) of antimicrobial resistance detected from fecal specimens and water from the Greater Gombe Ecosystem, Tanzania.

Group/Source	n	Sulfonamide (*sul1*, *sul2*)		Tetracycline (*tetA*, *tetB*)		Significance of *sul* to *tet* Frequency
		Positive	Frequency (95% CI)	Positive	Frequency (95% CI)	
**NHPs**						
Mitumba Chimpanzee	21	9	43 (23–66)	1	5 (0–26)	
Kasekela Chimpanzee	54	27	50 (36–64)	3	5 (0–16)	
Baboons	47	16	34 (21–49)	0	0 (0–95)	*p* < 0.54
All Chimpanzee	75	36	48 (36–60)	4	5 (1–13)	*p* < 0.05
ALL NHPs	122	52	43 (34–52)	4	0 (0–1)	*p* < 0.05
**Humans**						
Mitumba	32	26	81 (63–92)	8	25 (12–44)	
Kasekela	61	48	79 (66–88)	9	15 (1–27)	
Mwamgongo	94	65	69 (59–78)	9	10 (0–18)	
ALL HUMANS	187	139	74 (67–80)	26	14 (1–20)	*p* < 0.001
**Domestic Animals**						
Dog	7	5	71 (30–95)	1	14 (0–58)	Not evaluated
Goat	69	7	10 (5–20)	4	6 (0–15)	*p* = 0.37
Sheep	13	3	23 (1–54)	0	0 (0–28)	Not evaluated
ALL DOMESTIC ANIMALS	89	15	17 (10–27)	5	6 (0–13)	*p* < 0.001
Stream sites	21	4	19 (1–43)	0	0 (0–19)	
Pipe	1	0	0(0–95)	0	0 (0–95)	
ALL WATER	22	4	18 (1–41)	0	0 (0–60)	

**Table 2:** Proportion of sulfonamide and tetracycline genes identified from fecal specimens and stream sites in and around Gombe National Park, Tanzania.

		Frequency of Genes (n; (%)) Detected from Fecal Specimens by PCR							
		Number of Individuals Positive for Gene (Percentage)							McNemar’s *p*-Value Comparing *sul* to *tet* Gene Frequency *
Group/Source	n	*sul1 Only*	*sul2 only*	*sul1/sul2*	*tetA Only*	*tetB Only*	*tetA/tetB*	Negative	
**NHPs**									
*Mitumba Chimpanzee*	21	0 (0)	7 (33)	2 (10)	0 (0)	1 (5)	0 (0)	12 (57)	
*Kasekela Chimpanzee*	54	1 (2)	19 (35)	7 (13)	0 (0)	3 (6)	0 (0)	27 (50)	
*Baboon*	47	4 (9)	9 (19)	3 (6)	0 (0)	0 (0)	0 (0)	31 (66)	*p* > 0.05
All Chimpanzee	75	1 (1)	26 (35)	9 (12)	0 (0)	4 (5)	0 (0)	39 (52)	*p* < 0.001
ALL NHPs	122	5 (4)	35 (29)	12 (10)	0 (0)	4 (3)	0 (0)	70 (57)	
**Humans**									
*Mitumba*	32	1 (3)	15 (47)	10 (31)	0 (0)	8 (25)	0 (0)	4 (13)	
*Kasekela*	61	5 (8)	30 (49)	13 (21)	1 (2)	6 (10)	2 (3)	12 (20)	
*Mwamgongo*	94	7 (7)	30 (32)	28 (30)	1 (1)	8 (9)	0 (0)	29 (31)	
ALL HUMANS	187	13 (7)	75 (40)	51 (27)	2 (1)	22 (12)	2 (1.07)	45 (24)	*p* < 0.001
**Domestic Animals**									
*Dog*	7	0 (0)	2 (29))	3 (43)	0 (0)	1 (14)	0 (0)	2 (29)	
*Goat*	69	1 (1)	4 (6)	2 (3)	3 (4)	1 (1)	0 (0)	61 (88)	
*Sheep*	13	1 (8)	1 (8)	1 (8)	0 (0)	0 (0)	0 (0)	10 (77)	
ALL DOMESTIC ANIMALS	89	2 (2)	7 (8)	6 (7)	3 (3)	2 (2)	0 (0)	73 (82)	*p* < 0.001
Environmental									
*Stream sites*	21	0 (0)	4 (19)	0 (0)	0 (0)	0 (0)	0 (0)	17 (81)	
*Pipe*	1	0 (0)	0 (0)	0 (0)	0 (0)	0 (0)	0 (0)	1 (100)	
*ALL WATER*	22	0 (0)	4 (18)	0 (0)	0 (0)	0 (0)	0 (0)	18 (81)	

*p-values for statistical comparisons performed^.^

## Data Availability

Datasets generated during and/or analyzed during the current study can be directed to the corresponding author on reasonable request.
